# The Grand Challenges Discourse: Transforming Identity Work in Science and Science Policy

**DOI:** 10.1007/s11024-017-9332-2

**Published:** 2017-09-04

**Authors:** David Kaldewey

**Affiliations:** Forum Internationale Wissenschaft, University of Bonn, Heussallee 18-24, 53113 Bonn, Germany

**Keywords:** Grand challenges, Science policy, Identity work, Conceptual history, Sportification

## Abstract

This article analyzes the concept of “grand challenges” as part of a shift in how scientists and policymakers frame and communicate their respective agendas. The history of the grand challenges discourse helps to understand how identity work in science and science policy has been transformed in recent decades. Furthermore, the question is raised whether this discourse is only an indicator, or also a factor in this transformation. Building on conceptual history and historical semantics, the two parts of the article reconstruct two discursive shifts. First, the observation that in scientific communication references to “problems” are increasingly substituted by references to “challenges” indicates a broader cultural trend of how attitudes towards what is problematic have shifted in the last decades. Second, as the grand challenges discourse is rooted in the sphere of sports and competition, it introduces a specific new set of societal values and practices into the spheres of science and technology. The article concludes that this process can be characterized as the sportification of science, which contributes to self-mobilization and, ultimately, to self-optimization of the participating scientists, engineers, and policymakers.

**Electronic supplementary material:**

The online version of this article (doi:10.1007/s11024-017-9332-2) contains supplementary material, which is available to authorized users.

## Introduction

In the 20th century, science and politics collaborated to “solve problems.” In the 21st century, they collaborate to “tackle grand challenges.” This is, at least, the impression one gets when listening to scientists discussing research agendas and to science policymakers formulating funding schemes: “grand challenges” (GC) are everywhere. The rapid emergence and stabilization of the GC concept as well as its consequences for future developments have not yet been systematically explained or understood. The considerable career of the concept has been noticed, however, both by science and technology studies (STS), and by science, technology, and innovation (STI) policy studies. Scholars from STI policy studies have asked, for example, whether the GC concept marks a new paradigm in STI policy, or whether it is old wine in new bottles (Cagnin et al. 2012; Foray et al. 2012; Kallerud et al. 2013; Kuhlmann and Rip 2014; Hicks 2016; Ulnicane 2016). STS scholars, and, more recently, philosophers of science, have begun to examine how such a new policy rationale influences research trajectories, practices, methods, and, finally, scientific ethos (Winter and Butler 2011; Calvert 2013; Bos et al. 2014; De Grandis and Efstathiou 2016; Efstathiou 2016). Due to the analytical fuzziness of the GC concept, however, answers to these questions have remained vague. This article proposes to take a step back and examine the history and performativity of the concept itself, its tacit presuppositions, and the deep structure of the discourse in which it is embedded. In doing so, the paper prepares the ground for further studies and raises awareness and reflexivity about what is at stake when we frame the entanglements of science, technology, and society as “grand challenges.”

Making “grand challenges” the object of scholarly inquiry does not imply to sharpen the fuzzy concept, to reconcile contradictory expectations regarding its proper meaning and use, or, finally, to propose a workable definition. Instead, building on a social constructionist epistemology, which does not view language as representing reality, but as (co-)constitutive of social structures, the article analyzes and historicizes the GC concept as a “social fact.”^1^ The aim is to understand how and why actors in the field of science and science policy perform a GC discourse—or, the other way around, how they participate in a discourse which operates mostly behind their backs. The key question guiding the analysis is whether this GC discourse is or may become transformative in regard to the identity work of scientists and policymakers and in regard to their way of communicating with each other. The term “grand challenges” is thus interpreted as an *actor’s category*, and not employed as an *analyst’s category*.

The distinction between actor’s and analyst’s categories has to be kept in mind with regard to the various sources used in this article. On the one hand, the article builds on primary sources, in which actors talk or write about “grand challenges” (or related historical concepts) more or less consciously, and more or less strategically. On the other hand, the article refers to secondary sources, in which analysts in the field of STS and STI policy discuss the GC concept reflectively. However, these distinctions sometimes blur. Particularly if analysts aim at defining the GC concept in such a way that it helps to improve or transform science policy, then they act as players in the field and their papers may be read as primary sources. Take, for example, Gilbert S. Omenn’s presidential address at the 2006 annual meeting of the American Association for the Advancement of Science (AAAS)—an impressive paper delineating the career and the dissemination of GC lists in various fields. At the same time, however, Omenn strategically and reflectively uses the GC concept in order to demonstrate to the wider public the “added value of further major investments in research and development and education at a time of intense competition for funds” (Omenn 2006: 1696). Such amalgamations of scholarly reflection and strategic action are very common in the literature, while there are very few papers so far that discuss the role and effects of the GC discourse without at the same time arguing normatively and politically.

Analyzing “grand challenges” as discursive performances does not mean to deny that they at the same time indicate real problems. But these problems are not simply out there, they have a history, and their constitution as problems depends on what a society perceives as an issue to be addressed at a given time. The fact that specific phenomena are transformed into and explicated as “grand,” “global,” or “societal challenges” points to new modes of interaction between scientists, engineers, policymakers, and other stakeholders (Winter and Butler 2011; Calvert 2013; Hicks 2016; Kaldewey et al. 2017). Even if there is not yet a concluding answer to the question of how far the identity work of these actors has actually been transformed, the article presents evidence for an ongoing transformation. In other words, interpreting “grand challenges” as discursive performances, and, ultimately, assuming the existence of one cohesive GC discourse, does not mean to reduce the phenomenon to a mere rhetoric of research funding or to a problem of communicating science and technology to the public. Rather, the GC discourse is embedded in diverse institutions, and becomes visible not only in language, but, for example, in funding programs, organizational structures, and academic publishing practices. In an Appendix to this article (Online Supplementary Material), this correlation of conceptual framing and performative efficacy will be illustrated with regard to different communication contexts, such as science policy, higher education, and scientific publications. The Appendix furthermore points to the need for future studies that assess empirically how the GC discourse in these contexts not only transforms the identity work of the actors involved, but also affects their behavior.

Methodologically, the two main parts of this article build on conceptual history and historical semantics (for an overview, see Olsen 2012; Pernau and Sachsenmaier 2016; Müller and Schmieder 2016). The intention is to analyze “grand challenges” in the same way political philosophers and historians have analyzed contested political concepts, such as “freedom,” “democracy,” or “revolution.” The two parts follow an onomasiological and a semasiological perspective, respectively—a distinction that is commonly used in linguistics and semiotics (Baldinger 1998; Koch 2001) and that has been adapted for conceptual history by Reinhart Koselleck (1978: 30; see also Richter 1995: 47–48). The onomasiological approach assumes that there is a given phenomenon or idea that has been described with different terms in the course of history and in different contexts. To analyze the GC discourse onomasiologically, therefore, means to inquire those concepts that have been used in changing historical contexts to describe phenomena similar to those that are today framed as “grand challenges.” In other words, the onomasiological perspective indicates how we perceive, frame and address problems at a given time. Comparing such historical variations reveals a general cultural shift that can be summarized as a gradual substitution of “problems” by “challenges.” In contrast, the semasiological approach examines what a given term denotes in different contexts and how its meaning changes over time. Thus, a semasiological analysis of the GC discourse deals with the semantic field circumscribed by the literal terms “challenge,” “challenger,” and “grand challenges.” This analysis reveals that the semantics of the GC discourse is rooted in the sphere of sports and competition, and thus introduces a specific new set of societal values and practices into the sphere of science and science policy. In the conclusion this process will be described as the sportification of science.

## Part I: From Problems to Challenges

**Fig. 1:**
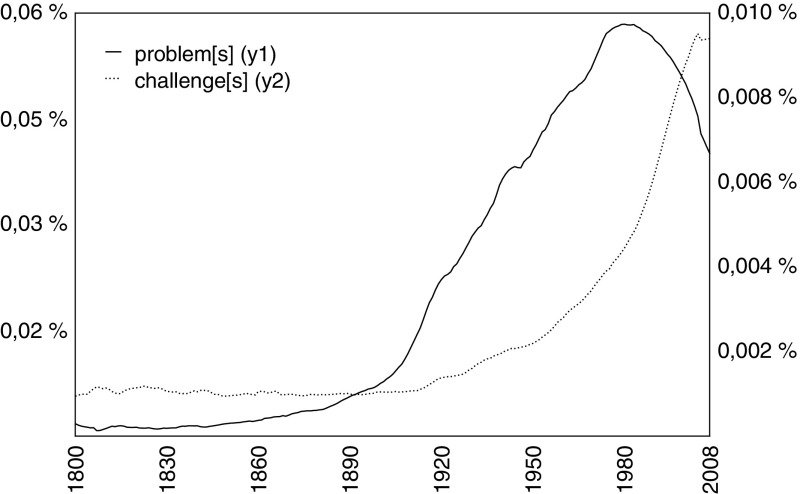
Relative frequencies of the terms “problem[s]” and “challenge[s],” extracted from Google Books Ngram Viewer, 1800–2008 (English corpus; case-insensitive; smoothing = 3)

**Fig. 2:**
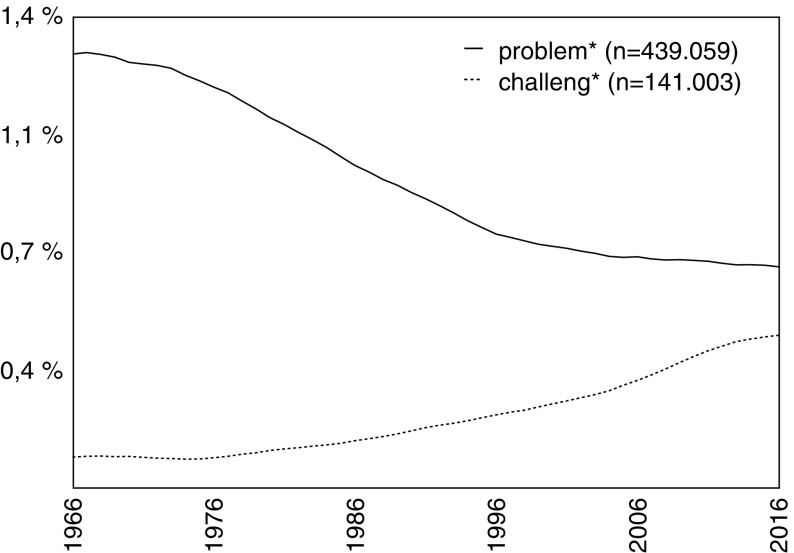
Relative numbers of publications in the Web of Science core collection that contain the terms “problem*” or “challeng*” in their title, 1966–2016 (data accessed February 14, 2017; total n = 53.773.845; smoothing = 3)

When Bill Gates announced the *Grand Challenges in Global Health* initiative at the World Economic Forum in Davos in January 2003, he said that he was inspired by the list of mathematical problems presented by David Hilbert in 1900 (Enserink 2003; Varmus et al. 2003). Since then, many authors have cited Hilbert as the inventor of the GC idea, although he himself did not use the term and despite the fact that his conception of “mathematical problems” differs considerably from the 21st century notion of “societal” or “grand challenges.”^2^ The word “challenge” (*Herausforderung*) is used neither in the original document (Hilbert 1900) nor in the English translation (Hilbert 1902). This subtle difference is relevant because it hints at a long-term semantic change over the course of the 20th century, a shift from framing the scientific endeavor in terms of “problems” to framing it in terms of “challenges.” This semantic shift can be visualized using the relative frequencies of the words “problem[s]” and “challenge[s]” in digital corpora, such as *Google Books Ngram Viewer* (see Fig. 1), and, regarding scientific communication, in the titles of publications listed in the *Web of Science* database (see Fig. 2).

Therefore, it is helpful to more closely examine the semantic field circumscribed by these terms. First, and in line with the Hilbert reference, some of the most prominent 20th-century philosophers and historians of science, such as Karl Popper (1999) and Thomas Kuhn (1970), stressed that “problem solving” or “puzzle solving” lies at the heart of normal science. However, these authors were careful not to equate normal scientific problems with what is implied in ordinary language when people talk about “problems”—namely, that something is somehow bothersome, dangerous, or even fateful. Such associations may explain to some extent why Kuhn preferred the more playful term “puzzle.” If we revisit the relevant passages in Kuhn’s work, two points are revealing. First, Kuhn links the idea of puzzles to the idea of a challenge that triggers the scientist’s motivation:

Bringing a normal research problem to a conclusion is achieving the anticipated in a new way, and it requires the solution of all sorts of complex instrumental, conceptual, and mathematical puzzles. The man who succeeds proves himself an expert puzzle-solver, and the challenge of the puzzle is an important part of what usually drives him on (Kuhn 1970: 36).

In the GC discourse, we often find similar notions linking the character of a challenge to the motivation and imagination of scientists (see also Winter and Butler 2011; Calvert 2013; Hicks 2016). In the words of Tom Kalil, a science policy advisor in the Obama administration, “Grand Challenges are compelling and intrinsically motivating.” As a consequence, Kalil continued, “people should be willing to devote a good chunk of their career to the pursuit of one of these goals” (Kalil 2012: 3). A similar argument was made by Bill Gates regarding the successful start of the *Grand Challenges in Global Health* initiative: “When scientists are given a chance to study questions that could save millions of lives, they eagerly rise to the challenge” (cited in BMGF 2005). This quote illustrates the idea that scientists’ intrinsic motivation must not be restricted to inner-scientific puzzles but can be channeled towards societal goals—such as saving lives.

A second aspect in Kuhn’s work, however, points in another direction. For Kuhn, puzzles are normally not at all “grand” or “socially important”; instead, he explicitly refers to the jigsaw puzzle and the crossword puzzle to clarify what he means by normal science puzzles. In line with this, he proposes a distinction between “puzzles” and “problems”:

It is no criterion of goodness in a puzzle that its outcome be intrinsically interesting or important. On the contrary, the really pressing problems, e.g., a cure for cancer or the design of a lasting peace, are often not puzzles at all, largely because they may not have any solution (Kuhn 1970: 36–37).

In this quote, Kuhn uses the expression “pressing problems” in regard to phenomena that today would be framed as “grand challenges.” Kuhn’s doubt that these problems have solutions reveals how cultural attitudes have shifted since his time. Whereas fifty years ago one could have spoken of problems that might not have a solution, protagonists of today’s GC discourse, particularly in the context of science policy, assume that GC are “ambitious but achievable” (Kalil 2012: 3), or, in another formulation, “feasible” given current capabilities (NRC 2001a: 2, 2004: 3; EC 2008: 38). The principle of feasibility is often complemented by the requirement of “measurable targets for success and timing of completion” (Kalil 2012: 3).

In contrast to the ideology of feasibility inherent in today’s GC discourse, discussions about the role of science in society in the 1960s and 1970s were much more prone to address critical, frustrating, or even aporetic problems. In his book *Inventing the Future*, Nobel laureate Dennis Gabor (1963: 3) identified a “Trilemma” consisting of three great threats of our civilization: “destruction by nuclear war,” “overpopulation,” and the “Age of Leisure,” i.e., the problem that people no longer know how to spend their free time. Although Gabor rated the third problem as the most difficult, his contemporaries mostly perceived the first as the ultimate danger. The likelihood that mankind may not survive the next few years was discussed in the scientific community in a manner that might be taken as an equivalent to today’s archetypical GC of climate change. In a programmatic *Science* article, John Platt delivered a typology of “crisis problems” that were classified relative to the likelihood of “total annihilation” (Platt 1969: 1118) and proposed a “large-scale mobilization of scientists” as the “only way to solve our crisis problems” (Platt 1969: 1115).

Another interesting case is a book by German political scientist and futurologist Ossip Flechtheim (1970), dealing with five challenges to which the coming discipline of futurology must respond: (1) The threat of war and the related danger of the physical extinction of humanity; (2) the nexus of overpopulation and hunger, especially in the Third World; (3) the exploitation and repression of human beings; (4) the degradation of the natural environment; and (5) the alienation and psychic deformation of the individual in industrialized societies. In a later book, Flechtheim updated this list, ending with “seven existential challenges” that he compared to the “seven deadly sins” that had been formulated by early Christian theologians (Flechtheim 1987: 95, my translation). Regarding the uncertain origin of the GC term, it is noteworthy that Flechtheim actually wrote about the “very big challenges of our epoch” (Flechtheim 1970: 311, my translation) and used the literal English term “challenges” in the German manuscript (Flechtheim 1970: 9, 311). Thus he may be one of the first writers to systematically substitute the semantics of “problems” with the semantics of “challenges.” Anticipating the feasibility rationale of the GC discourse, he paired the five challenges with “optimal solutions” (Flechtheim 1970: 313, my translation), making reference to specific futurological disciplines that are responsible for identifying these solutions: (1) The institutionalization of world peace, supported by peace research; (2) population control and ensuring nutrition security, supported by global development planning; (3) the humanization of the state and the democratization of society, backed by critical future studies; (4) the protection of nature with the help of environmental protection and planning agencies; and (5) the enhancement of humans to become creative creatures and creators, supported by pedagogics and the new discipline of “psychagogics.”

In the 1970s, the idea of existential but clear-cut problems was complemented and sometimes substituted by the conception of “wicked problems.” In their classic article, Horst Rittel and Melvin Webber (1973: 160) stressed that problems in the field of social policy and planning are “inherently different from the problems that scientists and perhaps some classes of engineers deal with.” Because wicked problems are ill-defined and dependent on political and ethical judgments, there are no clear criteria for their solution; they cannot be “solved” but at best “re-solved—over and over again.” Rittel and Weber distinguished this wickedness from the “tame” or “benign” problems of, for example, mathematics or the sciences. In these disciplinary contexts, “the mission is clear,” and there are “criteria that tell when *the* or *a* solution has been found” (Rittel and Webber 1973: 160, 162). Such criteria do not exist in the case of wicked problems. Whether one terminates or continues working on a wicked problem depends on external considerations, such as time, money, and patience.

Again, this demonstrates that the perception of societal problems in the 1960s and 1970s contrasts starkly with today’s definitions of GC, in which these problems are framed as feasible. However, there is an inherent contradiction in the GC discourse, because most of the common-sense grand challenges (e.g., climate change, energy security, demographic change) are obviously wicked and defy well-defined feasibility. The difference is one of framing: today, most participants in the GC discourse adopt the optimistic futurological stance sketched by Flechtheim and others and conceive of challenges as solvable.

Changing ideas about what constitutes a relevant problem and the question of whether science can and should tackle the existential problems that haunt society overall changed not only the way scientists conceived of their profession (self-descriptions) but also how the science system was perceived by politicians and other patrons (external descriptions). Starting in the late 1960s, one can observe a transformation of science policy discourses and a corresponding production of new semantics. Most of the emerging policy discourses at that time aimed at overcoming the basic/applied-distinction by introducing new categories of knowledge production. In the United States, terms such as “mission-oriented” or “mission-related,” which originally had been used to qualify the type of research conducted at federal agencies and laboratories, became popular for describing various basic research activities that are somehow directed to external goals (e.g., Kistiakowsky 1965; Waterman 1965; Abelson 1967). Some years later, the subcategory of “oriented basic research” was introduced in the second edition of the OECD Frascati Manual (1970). In Great Britain, Frederick Dainton (1971) proposed the concept of “strategic science” in a government green paper, which in turn was contested by Lord Rothschild (1971). Building on that dispute and criticizing the proliferation of such new categories, Rothschild (1972) supplemented a paper in *Nature* that polemically pointed to “forty-five varieties of research.” What most of these semantic variations had in common was that they conceived of research as simultaneously fundamental and of social or economic relevance. This basic idea also found resonance in the concepts of “interdisciplinarity” and “transdisciplinarity” that became popular in the 1970s.^3^

The emergence of these new concepts and their career in the 1970s and 1980s is of interest here because in the recent STI policy literature, the GC discourse is often conceived of as a reformulation of the idea of mission-oriented research (Gassler et al. 2008; Cagnin et al. 2012; Foray et al. 2012; Amanatidou et al. 2014; Wallace and Rafols 2015). Furthermore, when discussing the grand challenges of our time, many authors, including actors in the field of science policy and scholars producing the secondary literature, point to the legendary 20th-century “mission oriented” or “big science” research endeavors. The project most often recalled is the Apollo project, which was announced by John F. Kennedy in 1961 with the famous promise of “landing a man on the moon and returning him safely to the earth”—within 10 years.^4^ Another common reference is the Human Genome Project (HGP) initiated by several funding bodies in the United States in 1990, which was originally planned to take 15 years but finished earlier, in 2003.^5^ Both of these projects exemplify how a clear-cut, long-term mission could actually be fulfilled within an extended (but limited) time frame. Another big science program that is sometimes mentioned as an early example of a GC is the “War on Cancer” announced by Richard Nixon in 1971. However, because this venture failed (Faguet 2005), it is less suitable as a best-practice example. The “War on AIDS” would be a better example; at least it is generally assumed as having been more successful. Furthermore, it was linked explicitly with the idea of a challenge (although not yet framed as “grand”) in the late 1980s: “The American Medical Association (AMA) has accepted the challenge to be in the forefront of this war on AIDS” (Hotchkiss 1988: 282).

Nonetheless, in the 1990s and 2000s the metaphor of war seemed to lose its motivational power.^6^ The GC discourse avoids the language of “war” in favor of a more positive wording. Therefore, although diseases such as cancer and HIV/AIDS are beyond doubt serious problems, the *Grand Challenges in Global Health* initiative depicts them not as enemies but as intriguing challenges that can be tackled by sufficient will, motivation, and resources. Grant announcements were framed in the imperative form, such as “Create New Ways to Prevent or Cure HIV Infection,” and “Design New Approaches to Cure HIV Infection.”^7^ Obviously, the words used—“create,” “design,” “prevent,” “cure”—are part of a semantic field that lies far from the realities of death and suffering. Against this background, it is no surprise that representatives of the GC discourse are careful about pointing to historical precursors that are associated with war and conflict. The Manhattan Project, for example, would be an intriguing example for a GC, but it is only occasionally mentioned in the literature.^8^ Another obvious case would be Ronald Reagan’s Strategic Defense Initiative (SDI), which was announced in 1983 and was quickly dubbed the “Star Wars program” by the media. Nevertheless, it seems that both scientists and science policy practitioners avoid referring to this highly controversial program, which is inconsistent with the positive rhetoric of the GC discourse.

To summarize, the onomasiological perspective reveals how the semantic orientation of research in the 20th century shifted from “problems” to “challenges.” This transformation is visible in scientific books and papers, in popular writings of eminent scientists, as well as in science policy discourses. Several precursors of the GC discourse can be found in the 1960s and 1970s, with the introduction of new research categories such as “mission-oriented” and “problem-oriented” research, the emergence of “interdisciplinarity” and “transdisciplinarity” as new ideals guiding scientific practice, or the concept of “wicked problems.” Most of these semantic variations, however, did not find resonance in the science system as a whole (i.e., in all disciplines and research areas), and neither have they become global categories that transcend national science policies. What makes the GC discourse unique is that within a relatively short period of time, the GC concept has disseminated into nearly all disciplines, not only in the sciences, but also in the humanities and social sciences,^9^ as well as into a vast array of institutional contexts around the world. In the meantime, referring to “grand challenges” has become self-evident for scientists and policymakers alike.

## Part II: From the Sphere of Sports to the Sphere of Science and Politics

The onomasiological perspective illustrates how science and society have long communicated about those problems that seemed the most pressing at a given time. However, it was not until very recently that scientists and policymakers began to reframe these problems as challenges. To understand the implications of this semantic innovation, it is necessary to adopt a semasiological perspective and to analyze the context-dependent meanings of the literal phrase “grand challenges.” There is, however, not one single story. If we confine the analysis to the sphere of science and technology, then the origin of the phrase can be traced back to US science policy contexts in the late 1980s (see Table 1 for an overview of documents dealing with the concept).

**Table 1:** Early US science policy documents with explicit GC definitions

Context / Source	Definition
US Federal Coordinating Council for Science, Engineering, and Technology (OSTP 1987: 3)	A GC is a fundamental problem in science or engineering, with broad applications, whose solution would be enabled by the application of the high performance computing resources that could become available in the near future
US Federal Coordinating Council for Science, Engineering, and Technology (OSTP 1991: 56)	A GC is a fundamental problem in science and engineering, with broad economic and scientific impact, whose solution could be advanced by applying high performance computing techniques and resources
US National Research Council report on computer science and technology (NRC 1988a: 33)	As in other fields of scientific endeavor, in computer science and technology there are a number of GC worthy of long-term research support. Such challenges, if successfully met, would generate major advances in the field and create significant spin-offs in industry and government
US National Research Council report on environmental sciences (NRC 2001a: 2, 11)	GC are major scientific tasks that are compelling for both intellectual and practical reasons, that offer potential for major breakthroughs on the basis of recent developments in science and technology, and that are feasible given current capabilities and a serious infusion of resources
US Networking and Information Technology Research and Development Program (NITRD 2003: 2)	A GC is a long-term science, engineering, or societal advance, whose realization requires innovative breakthroughs in information technology research and development and which will help address our country’s priorities

**Fig. 3:**
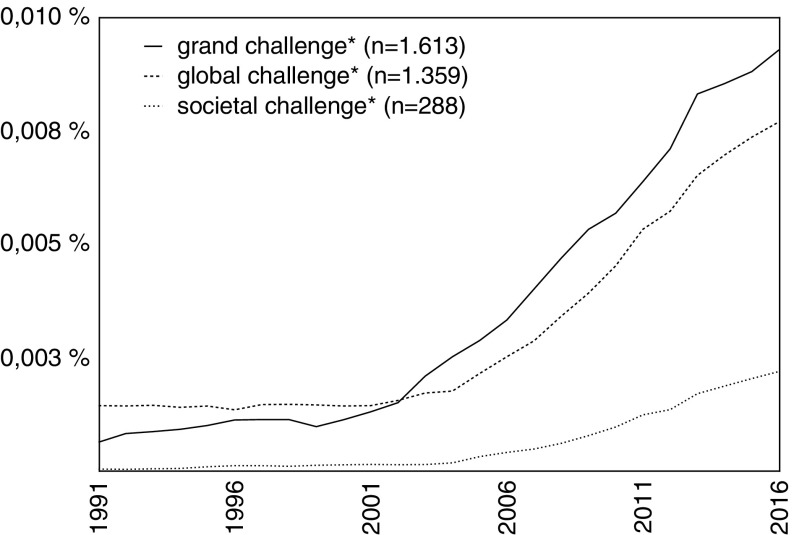
Relative numbers of publications in the Web of Science core collection that contain the terms “grand challenge*,” “global challenge*,” or “societal challenge*” in title, abstract, or keywords, 1991–2015 (data accessed February 14, 2017; total n = 37.345.793; smoothing = 3)

The first explicit definition is to be found in a 1987 report by the US Federal Coordinating Council for Science, Engineering, and Technology (FCCSET) that outlines the High Performance Computing and Communications (HPCC) program (OSTP 1987, 1991). Soon afterwards, the National Research Council (NRC 1988a, b) took up the GC term. At the same time, individual scientists such as Nobel laureate Kenneth Wilson (1988, 1989), and later Turing Award winner Raj Reddy (1988), used the GC concept to articulate their ideas for developing research agendas in the fields of computational sciences and artificial intelligence.^10^ Throughout the 1990s, the GC concept was nearly exclusively used in these epistemic communities.^11^ It was not until the 2000s that the GC discourse expanded in scope and disseminated to other disciplines and research fields.^12^ The career of the GC discourse in academic contexts, and, particularly, its momentum after the millennium, can be visualized using the *Web of Science* database. Figure 3 shows how the number of publications employing the GC concept has increased in the last 25 years.

The story of the invention of the GC concept in US science policy has been reconstructed in detail by Diana Hicks (2016). There is, however, a prehistory to these developments. In the course of the 20th century, there are several public discourses highlighting certain nationally framed challenges that have been perceived as threats in the United States and/or in Europe: the “Soviet Challenge” (1950s, 1980s), the “American Challenge” (1960s), and the “Japanese Challenge” (1980s) all have been important buzzwords at their time (see Fig. S1, Online Supplementary Material). As Hicks (2016: 24–26) notes, the competition with Japan was perceived as particularly pressing in the 1980s, and there is some reason to believe that it was this threat that was translated by Wilson (1984) into the GC discourse in the computational sciences. The NRC report *The National Challenge in Computer Science and Technology* pointed to Japanese developments several times (NRC 1988a: 26–36), and commenting on this report in *Science*, Mitchell Waldrop (1988) warned: “The United States is doing well, but the Japanese are gaining; perhaps we should accept some Grand Challenges.”

While the context of global competition between nation states is certainly important, there is still another, older, and probably even more relevant semantic baggage in the GC concept. This other story becomes visible when we analyze not only specific political and academic contexts, but furthermore the much older everyday meanings of the phrase “grand challenge” and its components. The term “challenge” has its origin in Middle English, where it was used in the sense of “accusation” (as a noun) or “to accuse” (as a verb). To be confronted with a challenge implied a demand to stand up against an accusation. The most salient form of such a challenge was the duel as an arranged combat between two individuals, traditionally noblemen. Following etymological dictionaries, this accusatory connotation died out in the 17th century. What remains today is the notion of someone participating “in a competitive situation or fight to decide who is superior in terms of ability or strength” (Oxford Dictionary of English, current online version 2015).

Since the 19th century, the term “challenge” has been particularly associated with the sphere of sports. In various disciplines, “challenge cups” have been institutionalized as specific forms of competition. Furthermore, the concept of a “world title challenge” evokes the older meaning of challenging an individual to a duel. There is also some evidence that the phrase “grand challenge,” which has never been common in everyday speech, has its origin in sports. In 1839, the “Grand Challenge Cup,” a men’s eight rowing competition, was initiated and institutionalized as the most prestigious event of the annual Henley Royal Regatta on the River Thames (see also Hicks 2016: 30–31). With few exceptions, that competition has continued through the present under the same name. Previously, the adjective “grand” had not been used as qualifier for “challenge,” and for some 150 years, the frequency of the English phrase “grand challenge” primarily related to this rowing competition.^13^ As illustrated in Figure S2 (Online Supplementary Material), the “grand challenges” quite suddenly left the sphere of sports in the 1980s and started their trajectory in the new context of US computer science and science policy. This origin of the GC idea in the sphere of sports is no coincidence and not without consequences. On the contrary, tracing the GC discourse in its formative phase from the late 1980s to the early 2000s, we find several discursive events that demonstrate a tight coupling of science and technology with the logic of sports.

First, in 1989, several prestigious Chinese universities initiated the “Challenge Cup Competition of Science and Technology,” a national, biennial event that is labeled the “Olympics” of science and technology among Chinese students. This competition has been supported by a range of scientific organizations and science policy institutions such as the Chinese Association for Science and Technology (CAST), the Communist Youth League, the Ministry of Science and Technology, and the Ministry of Education (Lu 2003: 77–78). The purpose of the National Challenge Cup is to motivate extracurricular activities and, more generally, “to uphold sciences, pursue truth, work hard, develop originality and take challenges.”^14^ Even if the organizers do not use the literal term “grand challenges,” this case is telling for two reasons. First, it shows how the logic of sports and competition can be translated and instrumentalized in higher education; and second, it hints to cultural shifts that are not exclusive to Western countries.

A second, more prominent case of using sports-like challenges to stimulate developments in science and technology is the annual international *RoboCup* competition, which took place for the first time in Nagoya, Japan, in 1997. Here, we find a direct link to the GC discourse as employed in US science policy in the late 1980s. The main figure behind the *RoboCup*, computer scientist Hiroaki Kitano, spent several years (1988–1994) as a visiting researcher at Carnegie Mellon University in the Unites States. In a paper written together with co-authors from different countries, Kitano et al. (1993) outline “Grand Challenge AI Applications” and refer explicitly to the GC definition of the HPCC program. Two years later, Kitano and several Japanese colleagues (1995) present the idea of using a “Robot World Cup” as a new “standard problem” for AI and robotics research. The following quote demonstrates the will to interlock the spheres of science, technology, and society:

Although it is obvious that building a robot to play a soccer game is an immense challenge, readers might wonder why we propose RoboCup. It is our intention to use RoboCup as a vehicle to revitalize AI research by offering a publicly appealing but formidable challenge. One of the effective ways to promote engineering research, apart from specific application developments, is to set a significant long-term goal. When the accomplishment of such a goal has significant social impact, it is considered a grand-challenge project (Kitano et al. 1997: 73).

Two decades later, it is clear that the *RoboCup* neither was nor is merely a leisure activity but is itself part of the academic game. As of 2016, the *Web of Science* core collection lists 563 publications that refer to the *RoboCup* competition in the title, abstract or keywords. Furthermore, the *RoboCup* soon became more than a soccer simulation. The initial *RoboCup Soccer League* (which itself has several subleagues) was supplemented by the *RoboCup Rescue Robot League* in 2001, in which robots are trained to help humans in hostile environments, particularly after earthquake disasters; followed by the *RoboCup Home league* in 2006, focusing on the role of robots in human interaction, and finally the *RoboCup Logistics League* in 2012. In these leagues, the aim is not only to win a competition but to solve real-world and societal problems.

The third event, the *DARPA Grand Challenge*, brings us back to US science policy contexts. Although it is in the United States that the GC discourse originally took off in the late 1980s, it is not until the 2000s that the semantics of sports and competition became institutionalized in new programs and noticed by a broader public. In January 2003, the Department of Defense announced the plan of a “Grand Challenge for autonomous robotic ground vehicles,” intended to spur technological development for military applications (DARPA 2003a, b).^15^ Teams of professionals and amateurs were invited to develop autonomous vehicles able to navigate an off-road course in the desert between Los Angeles and Las Vegas, with the winner promised a cash prize of $1 million. The inaugural challenge took place in March 2004; the course was 142 miles and the prescribed time limit 10 hours. However, none of the 15 machines that made it over the start line reached the goal, with the four most successful teams managing 5 to 7 miles before their cars dropped out. Although *Popular Science* magazine summarized the event as “DARPA’s debacle in the desert” (Hooper 2004), the agency itself was enthusiastic:

All across the nation, from garages to high schools, from universities to corporate laboratories, hundreds—perhaps thousands—of people worked on solving a problem important to the DoD. We had hoped that the Grand Challenge would excite many people, but it grew into something much, much bigger than anyone had imagined. The Congressionally authorized prize authority inspired many smart people who would not ordinarily work on a problem important to DoD, dedicating long days, nights and weekends toward finding a solution (Tether 2005: 8).

This statement by DARPA director Tony Tether, made while addressing the US Senate, builds on the rhetoric provided by the GC discourse, particularly the aspect of inducing motivation among researchers and inviting broad participation. In a press release Tether had explained that “we learned a tremendous amount today about autonomous ground vehicle technology,” and that even those vehicles that did not come very far “made it to the Challenge” (DARPA 2004). In other words, he highlighted not only the scientific relevance of the Challenge but also its character as a sports-like and participative event.

Consequently, the *DARPA Grand Challenge* was repeated, with the prize doubled to $2 million. The second competition took place in October 2005. This time, 5 out of 23 vehicles completed the course. The winning team was led by Sebastian Thrun, then the new head of Stanford’s Artificial Intelligence Laboratory (SAIL) and later responsible for Google’s driverless-car program. The event, the team, and the winning car, which was named Stanley, received not only media coverage, they were successful also in terms of scientific reputation. The *Journal of Field Robotics* published a highly cited paper with the title “Stanley: The Robot that Won the DARPA Grand Challenge” (Thrun et al. 2006).

Against the backdrop of these three cases, and in regard to the origin of the GC term in the sphere of sports, it is helpful to extend the semantic analysis of the term “challenge” with regard to the term “challenger.” Although this term is rarely explicitly used in the GC discourse, it is part of the semantic field evoked by this discourse. The dictionary contains two definitions of the word “challenger.” First, it is “a person who engages in a contest,” for example, a “championship challenger”; second, it is “a person who disputes the truth of or places themselves in opposition to something,” for example, a “challenger of authority” or a “challenger of campus orthodoxy” (Oxford Dictionary of English, current online version 2015). In other words, there are challengers in the sphere of sports and challengers in the sphere of truth. The term invites us to draw conceptual analogies between these different societal spheres. Furthermore, in the history of science and technology, “Challenger” has been a popular name for sea vessels, air- and space-craft, and land vehicles. Most prominent among these is the “Challenger Expedition” (1873–1876) organized by the Royal Society and named after the former Royal Navy Corvette HMS Challenger, which was turned into a research vessel. A century later, in memory of this mission, NASA’s second space shuttle was given the name Challenger. The history of this space shuttle, however, ended after only three years in the tragic accident of 1986. This catastrophe reminds us that if challenges are actually “grand,” then failure is an option (Pinkus et al. 1997). It is all the more surprising that at the very same time, the GC discourse took off in US science policy, conveying all of the positive connotations of feasibility mentioned above while completely ignoring the dark sides and risks of GC projects.

In summary, there is both a semantic baggage and a performative history in the GC discourse. Although the GC discourse seems to have become more autonomous since 2003, there is reason to believe that the semantics enshrined in the GC discourse tacitly introduce aspects of the logic of sports into the science system. This is particularly noteworthy when we compare the GC discourse to more traditional academic self-descriptions and science policy concepts: “pure science” originally had a religious and moral connotation (see Herzig 2005; Shapin 2012); “applied research” mostly refers to industry and business (see Johnson 2008; Lucier 2012); and labels such as “problem-oriented research,” “interdisciplinarity” and “transdisciplinarity” have a decidedly political tone (see Strohschneider 2014; Ledford 2015). All of these concepts translate the logic of specific societal spheres (religion, the economy, politics) to make sense of the practices and goals of scientific research. The GC discourse now discloses a new reference point for how we talk and think about science, technology, and their social embeddedness: the logic of sports and competition, leading to self-mobilization and, ultimately, to self-optimization of the participating scientists and engineers.

## Conclusion

Following conceptual history, language in general and contested concepts in particular are both “indicators” and “factors” of social and political change (Koselleck 2004: 251; see also Olsen 2012: 171). This is obvious with respect to political concepts—such as “freedom,” “democracy,” or “revolution”—that do not simply describe what happened in the course of history but were and are used by historical actors to make political change happen. The same is true for concepts that are less visible in public discourse but all the more relevant in the social system of science, as well as in the trading zones between science, technology, and politics. However, it is quite difficult to assess whether the GC discourse simply indicates a changing relationship among science, technology, and society, or whether it furthermore functions as a factor that effects these very changes. Both parts of this article, one arguing onomasiologically, the other arguing semasiologically, have made visible that ideas about the role of science and technology in society, as well as narratives about what it means to be a scientist are shifting over time. This does not imply that the GC discourse actually triggers this transformation, but there is enough evidence to conclude that semantic and structural shifts at least are co-produced.

The first part of this paper reconstructed a long-term conceptual shift in how the scientific endeavor is framed. Whereas 20th-century science policy discourses were dominated by reference to “problems,” we now observe an increasing substitution of “problems” by “challenges.” This subtle semantic difference reveals how cultural attitudes have shifted in the last few decades. While it was common in the 1960s to talk about “great dangers,” “pressing problems,” and “crisis problems,” the GC discourse establishes a more positive wording: problems, conceived as challenges, are “compelling,” “energizing,” and by definition, “feasible.” However, there is reason to doubt whether the likelihood of successfully addressing today’s grand challenges is actually higher than the solutions to those problems that figured most prominently in public and scientific discourse half a century ago. Compare, for instance, the threat of nuclear war with today’s archetypical challenge of climate change. It is far from certain that scientific progress can impede global warming; thus, perhaps the trend will be to live with it, similar to how nations learned to live with the threat of nuclear annihilation during the Cold War.

The second part of this paper traced the historical emergence and meaning of the literal phrase “grand challenge.” The semasiological analysis revealed that the GC discourse has its roots in the sphere of sports. The science system responds to this discourse by enacting forms of competition that are following the logic of sports, thereby building on the self-mobilization and self-optimization of its participants. A telling case, as elaborated in detail above, are events such as the *RoboCup* or the *DARPA Grand Challenge*. Listening to the actors organizing and legitimizing such competitions, we find an Olympics-styled rhetoric: “The important thing is not winning but taking part.”^16^ This subversively counters the ideology of feasibility we often find in political definitions of GC. Taking this language seriously, we might conclude that science is not just, as often asserted by STS researchers, “politics by other means”^17^ but is also “sports by other means.”

To conclude, a surprising effect of the GC discourse is what may be characterized as the *sportification* of science. An open question then is how this process of sportification relates to other ways in which science and its societal environment are coupled. For example, Peter Weingart has systematically distinguished influences of the economy, of politics, and of the media, and characterized them as “commercialization,” “politicization,” and “medialization,” respectively (Weingart 2001). Following Weingart, all “these trends point to a loss of distance between science and society” (Weingart 2002: 706). They are, in other words, not necessarily, but potentially threatening the autonomy and efficacy of science. In regard to the sportification of science, future research is needed to assess its effects for the practice and quality of scientific research. However, there is a crucial difference in the process of sportification when compared to, for example, commercialization or politicization. In the latter cases, the assumption is that powerful actors somehow intrude the science system in order to enforce their respective interests (Schimank 2015). In the case of sportification, in contrast, it would not make sense to assume that the system of sports is interested in or profits from the sportification of science. The argument of this paper is a different one. What has been shown is that scientists, scientific institutions, as well as policymakers are able to translate and utilize the semantics of other societal spheres. Traditionally, their self-descriptions were affected by the semantics of religion (e.g., “truth”), politics (e.g., “practice”), and the economy (e.g., “innovation”), while today, self-descriptions additionally adapt the semantics of sports (“grand challenges”). At this point, the notion of identity work is helpful to expound that such adaptations can be transformative—actors’ identities are not separable from actors’ practices. Finally, it is important to note that sportification is not necessarily a threat to the integrity of science. Rather, understood as a new semantic horizon, it may enable identity narratives that move creatively beyond the old narratives, particularly beyond the “tired categories” of basic and applied research (Hicks 2016: 39) and the related linear model of innovation. Using the semantics of sports, scientists, engineers, and policymakers may internalize societal values that are different from those that were influential in the past. It remains to be seen how deeply these semantics will transform the way we conduct scientific research.

## Electronic supplementary material

Below is the link to the electronic supplementary material.

Supplementary material 1 (PDF 137 kb)

Fig. S1 Relative frequencies of the compounds “American challenge[s],” “Japanese challenge[s],” and “Soviet challenge[s],” extracted from Google Books Ngram Viewer, 1920–2008 (English corpus; case-insensitive; smoothing = 3) (PDF 87 kb)

Fig. S2 Relative frequency of the compound “grand challenge cup” compared to the relative frequency of the compound “grand challenge,” extracted from Google Books Ngram Viewer, 1800–2008 (English corpus; case-insensitive; smoothing = 3) (PDF 85 kb)

## Footnotes

1 For similar research strategies see, for example, Benoît Godin’s (2006) analysis of the linear model, which, “with the help of statistics and methodolgocial rules […] became a social fact,” or Désirée Schauz’ (2014) extensive analysis of “basic research” as a key concept of science policy that can be examined by means of historical semantics.

2 Actually, Bill Gates was not the first linking David Hilbert to the GC idea (for earlier references, see Bedau et al. 2001; Green and Newth 2001). However, as Gates pushed the GC discourse in 2003, he also established an origin myth: the story has been copied numerous times.

3 These concepts have resonated not only in science policy contexts, but were adopted by various scientists that aimed for a closer alignment of science and society. Erich Jantsch (1972: 102), one of the earliest protagonists of this new discourse, aimed at “enhancing society’s capacity for continuous self-renewal.” William D. McElroy (1977: 269–270), at that time chancellor of the University of California, San Diego, stressed that universities need a stronger focus on interdisciplinary and “problem-oriented research” in order to “adjust to society’s needs.”

4 It was probably the science journalist Ivars Peterson (1988) who first drew the analogy between the “dramatic challenge” of the Apollo project to the emerging GC discourse in the field of supercomputer research. The analogy is reiterated by Kitano et al. (1997: 74), and later by many others.

5 The decoding of the human genome was framed as a GC long before the actual HGP turned out to be successful (Reddy 1988: 17; OSTP 1991: 7). After 2003, it was the now accomplished mission that was used as a GC best practice example by several authors (e.g., Collins et al. 2003: 835).

6 The metaphor was prominently instrumentalized by Vannevar Bush (1945), for whom the “war against disease” was a crucial argument for the continuation of federal science funding after World War II.

7 See www.grandchallenges.org/Explorations/Pages/AllTopics.aspx (retrieved March 2, 2017) for a complete list with topics of the GC Explorations program starting in 2008.

8 Omenn (2006: 1703) points to “Franklin Roosevelt’s assignment to develop and test a nuclear bomb in the Manhattan Project before the Nazis did so.” The Nazi reference in this quote explains that the project, after all, was a good thing. Alexander (2008) uses a similar affirmative formulation. A more critical alignment between the Manhattan project and the GC idea is made by Cech (2012: 90).

9 Recent publications discuss GC in fields such as mathematics education research (Stephan et al. 2015), organization studies (Ferraro et al. 2015), social work (Uehara et al. 2013), anthropology (Nolan 2013), and archaeology (Kintigh et al. 2014)—to name but a few examples.

10 Given this origin of the concept, it is not surprising that until today, computer and information sciences have been especially prone to the GC discourse. Scientists in a variety of related fields produced GC lists for their respective disciplines: For example, spoken language systems (Makhoul et al. 1990), medical informatics (Sittig 1994), complexity research (Green and Newth 2001), computing education (McGettrick et al. 2004), information systems (Winter and Butler 2011), cyberinfrastructure (NSF and ACCI 2011), and business and information systems engineering (Mertens and Barbian 2015). A broad and ambitious agenda for computing research in general has been presented by Hoare and Milner (2005).

11 There are, however, some early references to GC in the field of environmental research (Brown 1994), in ocean and polar sciences (ECOPS 1994; Hempel 1996; Prandle 1997), in geophysical research (Lyons 1998; Raeder et al. 1998), and in regard to educational problems (Ehrmann 1999).

12 The NRC, for example, began to introduce the GC terminology in new disciplinary contexts, such as the environmental sciences (NRC 2001a), physics (NRC 2001b), earthquake engineering (NRC 2004), and biology (NRC 2009).

13 Scanning the Google books corpus, there are only three appearances of the compound “grand challenge” before its becoming the name of the “Grand Challenge Cup” regatta. The first appearance is in a French-English version of the works of Molière, as a translation of the French “grand défi,” indicating that the French expression is more common in the 18th century. Furthermore, there are two theological texts which use the compound “grand challenge,” however, again not in a systematic or terminological manner.

14 Little information about this competition is available in English; the official website is in Chinese (http://www.tiaozhanbei.net). The quote is taken from documentation of the 13th National Challenge Cup Contest, which was held in 2013 at Soochow University, at the Website of Hong Kong Baptist University (HKBU) (http://sa.hkbu.edu.hk/lqc/challengecup, retrieved November 15, 2015).

15 The background of this initiative is a Congressional mandate from 2001 aiming to make one-third of the Armed Forces’ ground vehicles unmanned by 2015 (Tether 2005: 7). The schedule at DARPA’s archived original website (http://archive.darpa.mil/grandchallenge04/schedule.html, retrieved March 3, 2017) says that the event is announced July 30, 2002. However, the first press releases accessible are dated January 2, 2003 (DARPA 2003a) and February 22, 2003 (DARPA 2003b). What is worth mentioning in view of these dates is that the former release predates the announcement of Bill Gates’ *Grand Challenges in Global Health* on January 26, 2003 (BMGF 2003), by a few weeks only. The fact that neither of the parties refer to each other in their announcements is not surprising, but interesting all the same: Two very different programs, one decidedly military in character, the other decidedly civilian, use the same rhetorics to frame their goals.

16 This famous phrase was part of a speech given in London in 1908 by Pierre de Coubertin, founder of the International Olympic Committee. The statement continues: “What counts in life is not the victory but the struggle; the essential thing is not to conquer but to fight well.”

17 The phrase has been popularized by Bruno Latour. For a detailed analysis with more references, see Brown (2015: 11–12).
