# Supplementary material for: The Grand Challenges Discourse: Transforming Identity Work in Science and Science Policy
Source: Minerva. 2017 Sep 4;56(2):161–82. doi: 10.1007/s11024-017-9332-2 (PMC5948272; doi:10.1007/s11024-017-9332-2)
Supplement: Supplementary file 1 — Supplementary material 1 (PDF 137 kb) [file 11024_2017_9332_MOESM1_ESM.pdf]

Minerva Online Supplementary Material
DOI 10.1007/s11024-017-9332-2

Appendix: The Performative Efficacy of the Grand Challenges Discourse

David Kaldewey

©The Author(s) 2017. This article is an open access publication

The article "The Grand Challenges Discourse: Transforming Identity Work in Science and Science Policy" focused on how the GC discourse evolved historically, how it disseminated in science policy contexts, and how it was picked up by individual scientists and scientific communities. The hypothesis put forward against this background was that the GC label has the potential to transform the identity work of scientists and policymakers. Furthermore, the assumption was made that there is a performative dimension in the GC discourse, implying that the discourse not only affects the identity work of actors in the field, but also their actual behavior. In other words, and regarding the sportification of science described in the conclusion, a not yet answered question is whether, to what degree and how exactly scientists and policymakers act according to the logic of sports and competition that comes with the GC discourse. For reasons of space, the article had to stop at the descriptive level, leaving the analysis of performative effects open for future studies. However, it is possible to at least point to some empirical observations that indicate performative effects at different levels, and that have informed the arguments presented so far. This appendix brings together three examples of how the GC discourse changes not only self-descriptions, but also actual programs, structures, and practices in three different communication contexts: (1) science policy, (2) higher education, and (3) scientific journals. As these cases have not been analyzed systematically and empirically, more research is needed to corroborate the assumption of the performative efficacy of the GC discourse.

David Kaldewey
Forum Internationale Wissenschaft, University of Bonn, Heussallee 18-24, 53113 Bonn, Germany
E-mail: kaldewey@uni-bonn.de

Appendix 1: Grand Challenges and Science Policy

In 2009, the Obama administration issued their *Strategy for American Innovation*, which proposed to "harness science and technology to address the 'grand challenges' of the 21st century" (White House 2009: 22). In the following years, two updated versions of this report were published (White House 2011; 2015). During that time, the strategy evolved from simple to-do lists to a new and ambitious policy tool (Hicks 2016: 31-34). Between 2013 and the end of the Obama administration in January 2017, the Office of Science and Technology Policy (OSTP) displayed on its Website both a definition and a set of "grand challenges" that were funded by diverse US government institutions, such as the National Institutes of Health (NIH), the Defense Advanced Research Projects Agency (DARPA), the National Science Foundation (NSF), the Department of Education (DOE), the National Aeronautics and Space Administration (NASA), and the US Agency for International Development (USAID). Those funding programs aimed at transforming the identity work of scientists and engineers, particularly in regard to collaborative research ("all hands on deck"). Furthermore, the Obama administration's GC programs put high value on research fields that promised major impact on society and the economy. However, to assess the structural relevance of the GC discourse in US federal science policy, one would have to examine more systematically whether the administration actually shifted their focus away from more traditional funding schemes.

On the other side of the Atlantic, the GC discourse has become a crucial element in EU science policy. After 2000, the term "societal challenges" appeared occasionally in documents of the European Commission, but it is not until 2008 that an expert group prominently used the GC idea as the main rationale for the *European Research Area* (EC 2008). Shortly after, in the so-called *Lund Declaration* (2009: 40), a wide coalition of stakeholders and representatives from science policy, industry, and research organizations proposed that "European research must focus on the Grand Challenges of our time moving beyond current rigid thematic approaches." In 2011, the European Commission launched its eighth framework program for research and innovation under the name *Horizon 2020*. What distinguishes this program from its predecessors is that it defines "tackling societal challenges" as a third priority alongside the traditional goals of "generating excellent science" and "fostering industrial leadership" (EC 2011: 7). From a total budget of 77 billion euros, 29.7 billion have been dedicated to this new rationale (EC 2013). Given these numbers, there is no doubt that the EU commission does influence to some degree what kind of collaborative research is possible and rewarding in the European Research Area. However, for the Commission, the GC discourse is only one strategy among others in the STI policy game. The prestigious European Research Council (ERC), which is devoted solely to "excellence," and, ultimately, to what used to be called basic research, can be interpreted as an institution counterbalancing the performative effects of

the GC discourse. The way funding is (re-)distributed in the next framework program will be a first indicator of whether the GC discourse continues its triumphant success at the supranational level.

To summarize, several observers today perceive the GC discourse as a "consolidated trend in science policy" (De Grandis and Efstathiou 2016). As a consequence, and compared to the other two contexts presented in the next two subsections, the science policy context is exhaustively discussed in the literature (see, particularly, Kallerud et al. 2013; Kuhlmann and Rip 2014; Hicks 2016; Ulnicane 2016). This secondary literature so far mostly focuses on US federal and EU supranational science policy programs. The GC concept, however, is also used by international organizations such as the OECD (2010, 2012), as well as in various national contexts beyond the West, such as China and India (Hoareau McGrath et al. 2014). Furthermore, the GC discourse is not restricted to science policy in the narrow sense of national and supranational governance. There are different kinds of GC initiatives by non-governmental actors. A prominent example that has occasionally been mentioned in this article and that is very well documented in the secondary literature is the *Grand Challenges in Global Health* initiative (Enserink 2003; Varmus et al. 2003; Matthews and Ho 2008; Brooks et al. 2009; Verma 2009; Hicks 2016: 27-29). Other important instantiations of the GC discourse are to be found in engineering and scientific societies (NAE 2008; Royal Society 2011), which have been influential in terms of public visibility, but have not received much attention in the secondary literature (see, however, Cech 2012; Hicks 2016: 30).

Appendix 2: Grand Challenges and Higher Education

In 2007, Princeton University established a *Grand Challenges Program* that focuses on the issues of climate and energy, development, and health. A glossy pamphlet asks the reader to "imagine a world in which the brightest minds work together to solve humanity's most pressing environmental problems, a transformative world that expands classroom learning beyond traditional academic and national boundaries" (Princeton University 2011). In 2008, University College London launched the *UCL Grand Challenges* initiative to bring together expertise "from across UCL and beyond [...] to address the world's key problems." Four such challenges are specified: global health, sustainable cities, intercultural interaction, and human wellbeing. Following the president of UCL, the program "has become fundamental to our mission" and enables to draw "leading researchers" and "talented students" to UCL (cited in UCL 2012: 1). In the meantime, many comparable programs have been launched at prestigious universities, particularly in the United States (e.g., University of California, LA; University of Colorado, Boulder; Georgia Tech; University of Minnesota; Indiana University), Great Britain (e.g., University of Exeter; Newcastle University), and Australia (e.g., University of Melbourne). A particularly radical approach has been proposed by Michael Crow, president of

Arizona State University, who aims to achieve a comprehensive reconceptualization of research and teaching "to seek solutions to the grand challenges associated with sustainability," the result of which will be the "New American University" (Crow 2010: 489; see also Crow and Dabars 2015).

Another way in which the GC discourse crystallizes in academia is via scholarship programs. Against the background of the "Grand Challenges for Engineering" report (NAE 2008) and the 2013 "Global Grand Challenges Summit" in London, sponsored by the Royal Academy of Engineering (UK), the National Academy of Engineering (US) and the Chinese Academy of Engineering, eight US engineering schools cooperated to organize the joint *Vest Scholarship Program* and further summit meetings. The goal of the program is to give select international graduate students "the opportunity to pursue world-changing ideas at top U.S. universities" (NAE 2013).

These examples demonstrate that the GC discourse is not restricted to research policy, but also gains relevance in global higher education policy. If this trend continues, and particularly, if the GC discourse in the long run should actually transform the curricula in at least some of the leading research universities, then we can assume strong effects regarding the identity work of both undergraduate and graduate students. It is at the university that the identity of young people is shaped and challenged. If early career scientists are socialized in interdisciplinary contexts, dealing with the grand challenges of our societies, then their future research trajectories may be different from those embedded in traditional disciplinary venues. However, there are also critical voices analyzing the GC discourse as a "new higher education ideology" (Vostal et al. 2011: 64). This critique argues for the humanist university tradition which may help "questioning the very premises behind the grand challenges" (Vostal et al. 2011: 76).

Appendix 3: Grand Challenges and Scientific Publications

So far, the examples indicate the GC discourse's relevance in regard to science policy and higher education. What is still open is the question whether the discourse also impacts scientific communication and research practices, and thus what Nowotny et al. (2001) call the "epistemological core of science." Again, in absence of empirical studies dealing with this question, we can only point to specific developments that may indicate such impacts. A first clue is the changing environment for scientific publications (Lawrence 2003; Franzen 2012). As publishing is crucial for every scientist to stay in the game, the assumption lies at hand that if journals expect scientists to address grand challenges in their research, this may significantly influence problem choice and research trajectories.

One telling case involves a series of editorials in the *Academy of Management Journal* (AMJ) between 2011 and 2014 in which the editors explain to potential authors how to publish in AMJ. They begin with the criterion of "significance" and propose the selection of a research topic that "confronts

or contributes to a grand challenge" (Colquitt and George 2011: 432). The editors also address "Non-US authors" and suggest that such authors can realize a particular benefit from working on grand challenges because they help "shift an overt emphasis on country context to a more interesting theoretical question or problem-oriented framing" (George 2012: 1024). Furthermore, the editors point to the importance of pursuing bold ideas: "Not all our studies understandably will be grand, nor will they all challenge conventional wisdom, but considering the relative importance and scale of a problem will likely make a study more relevant to managers, and make it more interesting for our readers" (George 2014: 2).

Beyond such examples at the micro level of journals' publishing strategies, it is conspicuous that several prominent new journals are no longer organized around specific research fields or disciplines; instead, they focus on challenges such as climate change (e.g., *Nature Climate Change*, issued October 2010) or energy security (e.g., *Nature Energy*, issued January 2016). Other new journals are even more ambitious: The self-stated objective of *Elementa: Science of the Anthropocene* (launched 2013) is that of of accelerating scientific solutions to the challenges presented by our new era (UCP 2016). Following this invitation, eminent scientists explain to the reader which grand environmental challenges have to be solved by 2050 to avoid the possible collapse of civilization as we know it (Barnosky et al. 2016: 3). It is not likely that this kind of apocalyptic rhetoric will become common sense in scientific communication, but if we see this case in the light of the other examples, we can summarize that the GC discourse actually impacts communication strategies of journals, editors, and scientists. Even if in many cases the effect is primarily a reframing of research agendas in terms of "grand challenges," this reframing may in the long run transform our ideas of what it means to be a scientist and how to cooperate with others in regard to the grand challenges we promised to tackle.

References

Barnosky, Anthony D., Paul R. Ehrlich, and Elizabeth A. Hadly. 2016. Avoiding Collapse: Grand Challenges for Science and Society to Solve by 2050. *Elementa: Science of the Anthropocene* 4(000094).

Brooks, Sally et al. 2009. Silver Bullets, Grand Challenges and the New Philantropy. *STEPS Working Paper* 24. Brighton.

Cech, Erin. 2012. Great Problems of Grand Challenges: Problematizing Engineering's Understandings of Its Role in Society. *International Journal of Engineering, Social Justice, and Peace* 1(2): 85–94.

Colquitt, Jason A., and Gerard George. 2011. From the Editors: Publishing in AMJ – Part 1. Topic Choice. *Academy of Management Journal* 54(3): 432–435.

Crow, Michael M. 2010. Organizing Teaching and Research to Address the Grand Challenges of Sustainable Development. *BioScience* 60(7): 488–489.

Crow, Michael M., and William B. Dabars. 2015. *Designing the New American University.* Baltimore: Johns Hopkins University Press.

De Grandis, Giovanni, and Sophia Efstathiou. 2016. Introduction – Grand Challenges and Small Steps. *Studies in History and Philosophy of Science Part C* 56: 39–47.

EC, European Commission, Directorate-General for Research. 2008. *Challenging Europe's Research. Rationales for the European Research Area (ERA).* Report of the ERA Expert Group, EUR 23326 EN200. Luxembourg: Office for Official Publications of the European Communities.

EC, European Commission. 2011. *Proposal for a Regulation of the European Parliament and of the Council establishing Horizon 2020 – The Framework Programme for Research and Innovation (2014–2020).* Brussels, 30.11.2011, COM(2011) 809 final.

EC, European Commission. 2013. *Factsheet: Horizon 2020 budget* (25 November 2013). https://ec.europa.eu/programmes/horizon2020/sites/horizon2020/files/Factsheet_budget_H2020_0.pdf. Retrieved March 6, 2017.

Enserink, Martin. 2003. Bill Gates Plans a Hit List, With NIH's Help. *Science* 299: 641.

Flink, Tim. 2016. *Die Entstehung des Europäischen Forschungsrates. Marktimperative – Geostrategie – Frontier Research.* Weilerswist: Velbrück Wissenschaft.

Franzen, Martina. 2012. Making Science News: The Press Relations of Scientific Journals and Implications for Scholarly Communication. In *The Sciences' Media Connection: Public Communication and its Repercussions* (Sociology of the Sciences Yearbook, Vol. 28), ed. Simone Rödder, Martina Franzen, and Peter Weingart, 333–352. Dordrecht: Springer.

George, Gerard. 2012. From the Editors: Publishing in AMJ for Non-U.S. Authors. *Academy of Management Journal* 55(5): 1023–1026.

George, Gerard. 2014. From the Editors: Rethinking Management Scholarship. *Academy of Management Journal* 57(1): 1–6.

Hicks, Diana. 2016. Grand Challenges in US Science Policy Attempt Policy Innovation. *International Journal of Foresight and Innovation Policy* 11(1/2/3): 22–42.

Hoareau McGrath, Cecile et al. 2014. *The International Dimension of Research and Innovation Cooperation Addressing the Grand Challenges in the Global Context. Final Policy Brief.* RAND Report, Prepared for the European Commission Directorate General for Research and Innovation. European Commission/RAND Europe.

Kallerud, Egil et al. 2013. Dimension of Research and Innovation Policies to Address Grand and Global Challenges. *NIFU Working Paper* 13/2013. Oslo.

Kuhlmann, Stefan, and Arie Rip. 2014. The Challenge of Addressing Grand Challenges: A Think Piece on How Innovation Can Be Driven Towards the "Grand Challenges" as Defined Under the Prospective European Union Framework Programme Horizon 2020. https://ec.europa.eu/research/innovation-union/pdf/expert-groups/The_challenge_of_addressing_Grand_Challenges.pdf. Retrieved March 6, 2017.

Lawrence, Peter A. 2003. The Politics of Publication. *Nature* 422: 259–261.

Lund Declaration. 2009. Europe Must Focus on the Grand Challenges of our Time. In *New World – New Solutions. Research and Innovation as a Basis for Developing Europe in a Global Context.* Lund 7–8 July 2009, Sweden. Final Report, Appendix 2, 40–41. Lund: The Swedish EU Presidency Conference.

Matthews, Kirstin R.W., and Vivian Ho. 2008. The Grand Impact of the Gates Foundation. *EMBO reports* 9(5): 409–412.

NAE, National Academy of Engineering. 2008. *Grand Challenges for Engineering.*

NAE, National Academy of Engineering. 2013. *International Scholarship Focused on Global Grand Challenges Announced.* Press Release, March 13, 2013.

Nowotny, Helga, Peter Scott, and Michael Gibbons. 2001. *Re-Thinking Science: Knowledge and the Public in an Age of Uncertainty.* London: Polity Press.

OECD, Organisation for Economic Co-operation and Development. 2010. *The OECD Innovation Strategy: Getting a Head Start on Tomorrow.* Paris: OECD.

OECD, Organisation for Economic Co-operation and Development. 2012. *Meeting Global Challenges through Better Governance: International Co-operation in Science, Technology and Innovation.* Paris: OECD.

Princeton University. 2011. *Grand Challenges Program.* http://www.princeton.edu/grandchallenges/about/progress-report/gc_pamphlet.pdf. Retrieved August 19, 2015.

Royal Society. 2011. *Knowledge, Networks and Nations: Global Scientific Collaboration in the 21st Century.* RS Policy document 03/11, issued March 2011 DES2096. London.

UCL, Office of the Vice-Provost (Research). 2012. *UCL Grand Challenges. Progress to Date*. 2010/11 – 2011/2012. London.

UCP, University of California Press. 2016. *University of California Press to Publish Open Access Journal Elementa: Science of the Anthropocene*. Press Release, July 11, 2016.

Ulnicane, Inga. 2016. "Grand Challenges" Concept: A Return of the "Big Ideas" in Science, Technology, and Innovation Policy? *International Journal of Foresight and Innovation Policy* 11(1/2/3): 5–21.

Varmus, H. et al. 2003. Grand Challenges in Global Health. *Science* 302: 398–399.

Verma, G. 2009. Analysis of the Mass Media Coverage of the Gates Foundation Grand Challenges in Global Health Initiative. *Journal of Medical Ethics* 35(3): 163–167.

Vostal, Filip, Lorenzo Silvaggi, and Rosa Vasilaki. 2011. One-Dimensional University Realised: Capitalist Ethos and Ideological Shifts in Higher Education. *Graduate Journal of Social Science* 8(1): 62–82.

White House, Executive Office of the President, National Economic Council, and Office of Science and Technology Policy. 2009. *A Strategy for American Innovation. Driving Towards Sustainable Growth and Quality Jobs*. September 2009. Washington, DC.

White House, Executive Office of the President, National Economic Council, Council of Economic Advisors, and Office of Science and Technology Policy. 2011. *A Strategy for American Innovation. Securing Our Economic Growth and Prosperity*. February 2011. Washington, DC.

White House, Executive Office of the President, National Economic Council, and Office of Science and Technology Policy. 2015. *A Strategy for American Innovation.* October 2015. Washington, DC.
