# Supplementary material for: The Grand Challenges Discourse: Transforming Identity Work in Science and Science Policy
Source: Minerva. 2017 Sep 4;56(2):161–82. doi: 10.1007/s11024-017-9332-2 (PMC5948272; doi:10.1007/s11024-017-9332-2)
Supplement: Supplementary file 3 — Relative frequency of the compound “grand challenge cup” compared to the relative frequency of the compound “grand challenge,” extracted from Google Books Ngram Viewer, 1800–2008 (English corpus; case-insensitive; smoothing = 3) (PDF 85 kb) [file 11024_2017_9332_MOESM3_ESM.pdf]

Minerva Online Supplementary Material
DOI 10.1007/s11024-017-9332-2

The Grand Challenges Discourse: Transforming Identity Work in Science and Science Policy

Figure S2

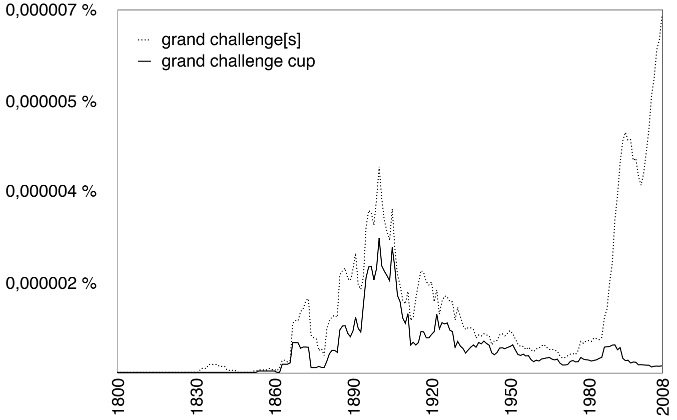

Fig. S2 Relative frequency of the compound "grand challenge cup" compared to the relative frequency of the compound "grand challenge[s]," extracted from Google Books Ngram Viewer, 1800–2008 (English corpus; case-insensitive; smoothing=3).

David Kaldewey
Forum Internationale Wissenschaft, University of Bonn, Heussallee 18-24, 53113 Bonn, Germany
E-mail: kaldewey@uni-bonn.de
